# 50 Metformin versus Insulin: Glucose Control and Safety in Burn Patients

**DOI:** 10.1093/jbcr/irad045.024

**Published:** 2023-05-15

**Authors:** Taylor Hallman, Steven Wolf, Georgiy Golovko, Juquan Song, Amina El Ayadi

**Affiliations:** The University of Texas Medical Branch, Galveston, Texas; The University of Texas Medical Branch, Galveston, Texas; The University of Texas Medical Branch, Galveston, Texas; The University of Texas Medical Branch, Galveston, Texas; The University of Texas Medical Branch, Galveston, Texas; The University of Texas Medical Branch, Galveston, Texas; The University of Texas Medical Branch, Galveston, Texas; The University of Texas Medical Branch, Galveston, Texas; The University of Texas Medical Branch, Galveston, Texas; The University of Texas Medical Branch, Galveston, Texas; The University of Texas Medical Branch, Galveston, Texas; The University of Texas Medical Branch, Galveston, Texas; The University of Texas Medical Branch, Galveston, Texas; The University of Texas Medical Branch, Galveston, Texas; The University of Texas Medical Branch, Galveston, Texas; The University of Texas Medical Branch, Galveston, Texas; The University of Texas Medical Branch, Galveston, Texas; The University of Texas Medical Branch, Galveston, Texas; The University of Texas Medical Branch, Galveston, Texas; The University of Texas Medical Branch, Galveston, Texas; The University of Texas Medical Branch, Galveston, Texas; The University of Texas Medical Branch, Galveston, Texas; The University of Texas Medical Branch, Galveston, Texas; The University of Texas Medical Branch, Galveston, Texas; The University of Texas Medical Branch, Galveston, Texas

## Abstract

**Introduction:**

Patients suffering from moderate-to-severe burns are known to experience significant metabolic perturbations, such as hyperglycemia, as the result of a systemic hypermetabolic response. Hyperglycemia in these patients is in part due to the development of peripheral insulin resistance. Glycemic control is important, as hyperglycemia in patients with moderate-to-severe burns is associated with increased morbidity and mortality. Insulin and metformin are the first lines of treatment, with previous studies showing metformin to be superior to insulin in reducing burn-induced hyperglycemia. We queried a large multi-institutional national database to investigate whether the use of metformin in burn patients is associated with improved glycemic control and morbidity/mortality outcomes compared to insulin therapy.

**Methods:**

We accessed TriNetX, a global health research network, and queried ICD-10 codes for burn injuries (T20-25, T30-32) across 54 participating health care organizations. We then identified cohorts of patients who were administered either metformin or insulin, but not both, within 1 week of burn. These cohorts were balanced using propensity score matching for age, gender, ethnicity, race, and type 2 diabetes mellitus and subsequently compared for the following outcomes: hyperglycemia (R73), hypoglycemia (E16.1, E16.2), lactic acidosis (E87.2), sepsis (A41), and death.

**Results:**

A total of 16,569 patients were given insulin within 1 week of burn. This patient cohort had a mean age of 54 years, with 60% being male, 67% White, and 19% Black or African American. In contrast, 7,308 patients received metformin within 1 week of burn, with a mean age of 56 years, 55% female, 70% White, and 16% Black or African American. Burn patients who received insulin within 1 week of burn showed a comparative increased risk of 3.009% (p < 0.0001) for the development of hyperglycemia compared to those who were given metformin within 1 week of burn (RR: 3.165, 95% CI: 2.433-4.117). Additionally, burn patients given insulin displayed an increased risk of 2.452% for development of hypoglycemia (p < 0.0001, RR: 4.555, 95% CI: 3.317-6.255), 3.078% for development of lactic acidosis (p < 0.0001, RR: 17.541, 95% CI: 10.003-30.76), 3.67% for development of sepsis (p < 0.0001, RR: 12.106, 95% CI: 7.898-18.557), and 7.422% for death (p < 0.0001, RR: 13.996, 95% CI: 10.212-19.182).

**Conclusions:**

Patients receiving insulin within 1 week of burn showed increased risk of poor glycemic control than those receiving metformin. Morbidity and mortality are also higher in those receiving insulin than those receiving metformin. Additional research is necessary to further understand these findings.

**Applicability of Research to Practice:**

These findings should encourage clinicians to appreciate the importance of glycemic control in burn patients, as well as recognize the importance of choosing the most appropriate pharmacologic agent for glycemic control.

